# Liver Robotic Surgery: A Review of Current Use and Future Perspectives

**DOI:** 10.3390/jcm14197014

**Published:** 2025-10-03

**Authors:** Vincenzo Schiavone, Filippo Carannante, Gabriella Teresa Capolupo, Valentina Miacci, Gianluca Costa, Marco Caricato, Gianluca Mascianà

**Affiliations:** 1 Department of Advanced Biomedical Sciences, University of Naples Federico II, 80131 Naples, Italy; vincenzo.schiavone@unina.it; 2 Department of Colorectal Surgery Unit, Fondazione Policlinico Campus Bio Medico, 00100 Rome, Italy; f.carannante@policlinicocampus.it (F.C.); g.capolupo@policlinicocampus.it (G.T.C.); valentina.miacci@policlinicocampus.it (V.M.); g.costa@policlinicocampus.it (G.C.); m.caricato@policlinicocampus.it (M.C.); 3 Department of Life Sciences, Health and Health Professions, Link Campus University, 00100 Rome, Italy

**Keywords:** colorectal surgery, liver surgery, robotic, colorectal metastasis

## Abstract

**Background:** Robotic liver surgery is emerging as a key advancement in minimally invasive techniques, though it still faces several challenges. Meanwhile, colorectal cancer (CRC) continues to be a leading cause of cancer deaths, with liver metastases affecting 25–30% of patients. These metastases significantly burden healthcare systems by raising costs and resource demands. **Methods:** A narrative literature review was performed, resulting in the inclusion of 14 studies in our analysis. Fourteen studies met the inclusion criteria and were analyzed with attention to patient characteristics, surgical details, perioperative outcomes, and reporting limitations. For consistency, simultaneous robotic-assisted resection (RAR) refers to cases in which the colorectal primary and liver metastasectomy were performed during the same operative session. **Results:** The 14 studies included a total of 771 patients (520 males and 251 females), aged between 31 and 88, undergoing simultaneous robotic-assisted resection (RAR). Most were affected by rectal cancer (76%) and unilobar liver metastases (82%). All surgeries using the DaVinci system are represented by 62% wedge resection and 38% anatomical (21.39% major and 16.61% minor). Patients’ BMI ranged from 19.5 to 40.4 kg/m^2^, the average blood loss was 181.5 mL (30–780), the median hospital stay was 7 days (range 2–28), and the mean operative time ranged from 30 to 682 min. Data on POLF (postoperative liver failure) are reported in two studies: Rocca et al., 1/90 patients; Marino et al., 1/40 patients. Biliary leak is reported in one case by Marino et al., while Winckelmans et al. reported a 2.6% incidence of biliary leak in the laparoscopic group and 3.4% in the robotic group. **Conclusions:** As research advances and new therapies emerge for colorectal liver metastasis (CRLM), surgery remains the mainstay of treatment. However, evidence is limited by small sample sizes, heterogeneous study designs, inconsistent reporting of perioperative chemotherapy, timing of surgery, metastasis localization, and complications. Robotic liver surgery has become a well-established technique and possibly represents the future for managing colorectal liver metastases. Further prospective and comparative studies with standardized outcome reporting are needed to define optimal patient selection and long-term effectiveness.

## 1. Introduction

Robotic liver surgery is increasingly being recognized as the next step in minimally invasive procedures and is currently undergoing significant advancement. Robotic liver resections have been described in the literature since the early 2000s. Robotic systems enable highly precise maneuvers through articulated instruments with seven degrees of freedom, while also eliminating hand tremors and providing an enhanced surgical view with detailed three-dimensional, high-definition imaging [1]. Its technology represents the most advanced form of minimally invasive surgery, though there are still some unresolved challenges regarding its implementation in clinical practice [2]. The robotic system provides a stable visual platform, eliminates physiological tremors, enhances surgical precision, and offers better ergonomics through a seated operating position [3]. Given these potential benefits, robotic assistance may be particularly well suited for complex liver procedures, which require delicate tissue handling, highly accurate intracorporeal suturing, and intricate parenchymal dissection—followed by the need for meticulous bleeding and bile leak control. Parenchymal transection remains one of the most technically demanding aspects [4]. Robotic hepatectomy is commonly used to treat liver tumors and has been shown to be a safe and feasible option for patients with large (≥5 cm) hepatocellular carcinoma. Giulianotti et al. first reported robotic-assisted laparoscopic hepatectomy [2]. Since then, medical centers around the world have shared their experiences with this technique [5]. To date, the lack of specialized instruments and the development of various techniques have prevented the establishment of a standardized surgical approach. Currently, there is an increasing use of ICG-fluorescence imaging in patients undergoing minimally invasive resections for colorectal liver metastases, aiming to improve radical resection rates and the accuracy of tumor margin identification. At the same time, colorectal cancer (CRC) remains one of the primary causes of cancer-related mortality, with liver metastases occurring in approximately 25–30% of patients [6]. The presence of metastatic disease in the liver places a considerable burden on the healthcare system, not only by driving up costs and expenditures but also by increasing the need for and use of medical resources [7]. Robot-assisted simultaneous colon and liver resections are becoming increasingly common as the use of robotic platforms grows in the surgical treatment of metastatic colon cancer. Nevertheless, this technique remains underexplored, with current evidence limited to case series. In this review, we provide an overview of recent advances in the surgical, robotic treatment of colorectal liver metastasis (CRLM).

## 2. Materials and Methods

### 2.1. Data Collection

The team of surgeons compiled a Microsoft Excel database that included demographic, clinical, surgical, and pathological data. For clarity, throughout this manuscript, the term “simultaneous robotic-assisted resection (RAR)” is used to indicate cases in which the colorectal primary resection and the liver metastasectomy were performed during the same operative session.

### 2.2. Literature Review

A narrative review of the literature was conducted. This review follows a narrative approach and is not intended as a systematic review. The aim was to synthesize the most relevant literature on surgical and robotic management of CRLM. The search was narrowed to articles between 2010 and 2025. We searched PubMed and Cochrane Library; studies were included if they were full-text articles, reviews, or case reports published in English involving human adult participants (>18 years) and addressed aspects of the diagnosis, management, or treatment of CRLM. Studies were excluded if they were in other languages, lacked a visible abstract, or were not relevant.

This research was conducted using the following terms: “robotic liver resection AND colorectal metastasis”. Single case reports were included. The search yielded 98 results. After removing 1 duplicate and excluding 25 because they were in other languages or lacked a visible text or abstract, 72 remained.

A total of 58 studies were excluded because they were not relevant to our focus, considering the topics covered (because, after the research was conducted through PubMed and Cochrane Library, even though they met the inclusion criteria regarding language and participants, we excluded from our review all studies that did not address the treatment of liver metastases from colorectal cancer); for example, at this stage, we excluded some studies on non-surgical treatments.

**Figure 1:**
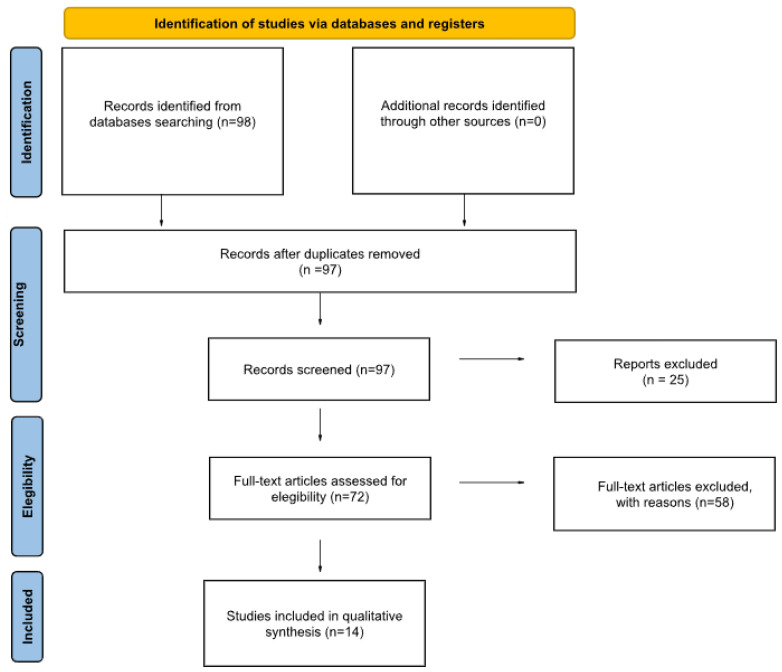
PRISMA flowchart diagram.

Finally, 14 texts were included in our review as reported in our PRISMA flowchart diagram [8], as shown in our figure (Figure 1).

## 3. Results

A total of 14 studies that included patients with CRLM who underwent RLR (robotic liver resection) were considered eligible for inclusion in our narrative review. Four of them were single case reports; one was a systematic literature review; one was a multicenter, prospective study; the others were all monocentric studies (five retrospective and three prospective). Four of them originated from Italy, two from the United States, two from Germany, one from Belgium, one from China, one from India, one from Brazil, one from Korea, and one from the Netherlands. The indices analyzed are presented in Table 1.

## 4. Main Outcomes

The 14 included studies analyzed ranged from single case reports to studies with hundreds of patients who underwent RAR, and the male sex was generally more prevalent. Among the included patients, the ages ranged from 31 to 88 years. The primary tumor site was prevalent in the rectum (76%). CRLMs were unilobar in 82% of patients.

Rahimli et al. [11] reported a prevalence of cirrhosis in 12 patients (10 Child A, 2 Child B), and hepatic steatosis was present in 14 patients, while hepatitis was reported as follows: 2 patients with hepatitis B and 1 with hepatitis C.

All procedures were performed with the da Vinci Surgical System (Intuitive Surgical, Mountain View, CA, USA) in its subsequent models. In six studies, RLR was performed using the new da Vinci Xi. Out of 771 patients, 478 underwent wedge liver resection (62%) and 293 received anatomical resections (38%). Among anatomical resections, major hepatectomy (more than three segments) was performed in 165 cases (56.3%), while the resection was classified as minor (three segments or fewer) in 128 (43.7%) cases. Among the included patients, the BMI ranged from 19.5 to 40.4 (kg/m^2^).

The median LOS was 7 days (range 2–28 days). Navarro et al. showed a series where there were no cases requiring conversion to open surgery. Robotic SCLR for colorectal cancer with liver metastases can be carried out safely, even when major liver resections are needed, particularly in specialized centers with experienced and well-trained teams [9]. The mean operative times ranged from 30 to 682 min. A longer operative time is considered to be a drawback of robotic procedures. This is due to the time needed to prepare and dock the robot, as well as to change instruments during the procedure.

Winckelmans et al. explored the economic aspects of robotic surgery—an especially relevant and widely discussed topic in the field—by comparing robotic liver resections with laparoscopic ones. They found that the expenses associated with surgical instruments and hospital stay were notably lower, whereas the costs linked to operative time were higher, in the robotic group [21].

Data on POLF (postoperative liver failure) are reported in two studies: Rocca et al., 1/90 patients; Marino et al., 1/40 patients. Biliary leak is reported in one case by Marino et al., while Winckelmans et al. reported a 2.6% incidence of biliary leak in the laparoscopic group and 3.4% in the robotic group, respectively.

Not all studies reported data on blood loss: 13 of them reported a median blood loss of 181.5 mL (30–780). Regarding the Clavien–Dindo classification, the complication rate was 19.2% grade I, 0.9% grade II, 8.5% grade III, 0.4% grade IV, and 0.2% grade V [23]. Regarding the timing of robotic surgeries, the approach was simultaneous with colorectal surgery in five studies and conducted in two steps in two studies; it was not specified in the remaining ones.

## 5. Long-Term Outcomes

Most of the studies did not report long-term outcomes. Navarro et al. [4] reported 12 cases followed for 47.1 months of disease-free survival (DFS) and 75.2 months of overall survival (OS).

Guadagni et al. [7], for 20 cases, reported a 1- and 3-year disease-free survival of 89.5% and 35.8%, respectively.

Pesi et al. reported that 4% of the patients (two) died. Local recurrence was diagnosed in five patients (10%), whereas distant metastases were observed in five patients (10%) [19]. Rocca et al. indicated that short- and long-term outcomes are superimposable compared to laparoscopy and RLR (robotic liver resection) [13].

## 6. Discussion

The global incidence of CRC has been rising with an annual increase of 3.2%, from 783,000 cases in 1999 to 1.8 million in 2020 [24]. Colorectal cancer (CRC) is the third-most common malignancy and the second-most common cause of cancer-related mortality worldwide. It is among the most common causes of cancer-related mortality, with liver metastases occurring in roughly 25–30% of diagnosed individuals. Around 15% of patients were found to have liver metastases at the time of diagnosis, and nearly half of all individuals with colorectal cancer are expected to develop liver metastases at some point during their lives [25]. Earlier studies indicate a variation in survival outcomes between patients with liver metastases originating from right-sided versus left-sided CRC, even though left-sided tumors tend to develop liver metastases more frequently [6].

The development of liver metastases from colorectal cancer is a prolonged and intricate process. It encompasses the persistence of the primary tumor, invasion into blood vessels, distant spread, and the eventual formation of metastases. The establishment of a supportive microenvironment prior to metastasis significantly facilitates tumor colonization and growth. During the progression of liver metastases in colorectal cancer, these events do not occur in a strict sequence but often simultaneously. The liver, which receives blood from the gastrointestinal tract, is the most common site for colorectal cancer to spread. It contains various resident cell types that contribute to the formation of a pre-metastatic niche. Some of these cells aid in tumor attachment and colonization, whereas others support tumor cell growth, modulate the immune environment, or promote the formation of new blood vessels [26]. Interventional oncology plays a key role in managing liver metastases from colorectal cancer by expanding the pool of patients eligible for surgery, offering potentially curative options to those who cannot undergo surgery, and enhancing survival outcomes in palliative care scenarios. While surgical removal remains the preferred approach when feasible, image-guided ablation—either alone or combined with surgery—has shown better survival outcomes compared to chemotherapy by itself. Selecting appropriate candidates—particularly those with tumors smaller than 3 cm— and ensuring ablation margins exceed 5 mm can help reduce local tumor recurrence. Techniques such as cryoablation may be effective in alleviating symptoms caused by painful lesions, while irreversible electroporation (IRE) offers a non-thermal option for tumors located near sensitive structures, such as bile ducts, where heat-based treatments are less suitable due to the heat sink effect. As treatment strategies continue to shift, more research is needed, particularly given the rising incidence of colorectal cancer in younger individuals [27]. When examining surgical options, robotic techniques have seen significant advancements. Laparoscopic surgery is now widely regarded as the standard approach for specific procedures, such as left-lateral sectionectomies and wedge resections of anterior liver segments. However, the exact role of robotic liver surgery remains under discussion, particularly in terms of long-term cancer-related outcomes. Robotic technology represents the most advanced form of minimally invasive surgery, though there are still some unresolved challenges regarding its implementation in clinical practice. Robot-assisted, minimally invasive surgery has already become a reality and is likely to be the future standard for many procedures. Conventional laparoscopy has several limitations, such as restricted movement, difficulty performing precise sutures, awkward surgeon positioning, and a lack of 3D visualization. Robotic surgery addresses many of these challenges and broadens the potential for minimally invasive surgery to benefit more patients [2]. Robotic surgery presents as a compelling minimally invasive alternative for liver procedures due to enhanced visualization and the use of highly articulated instruments [28]. Challenges such as longer operative times and the absence of tactile sensation have been noted, although these drawbacks have not been clearly demonstrated in clinical studies. On the other hand, according to Machado et al., robotic repeat hepatectomy is a viable and safe option when performed by skilled surgeons and may offer certain benefits compared to laparoscopic or open repeat liver resections [14]. Unique aspects of robotic procedures, including specific safety measures, must be established and reviewed at the start of each operation to prepare for possible emergency conversions. Although many liver surgeons have been slow to adopt this technique, robotic liver surgery continues to develop and gain acceptance in the field. Current data indicate that cancer outcomes following robotic liver resections are comparable to those seen with open or laparoscopic techniques for both hepatocellular carcinoma and colorectal liver metastases, with additional benefits noted in cirrhotic patients and those requiring repeat operations [29]. Achieving optimal outcomes and maintaining high levels of patient safety require targeted training in both hepatobiliary and general minimally invasive surgical skills to successfully navigate the learning curve. More recent studies have further demonstrated lower conversion rates and fewer complications than those observed with laparoscopic hepatectomy. For parenchymal transection, several techniques have proven effective in a robotic setting, including the clamp-and-crush method with bipolar forceps, the use of sealing devices or ultrasonic shears, and hybrid approaches combining ultrasonic surgical aspirator with robotic forceps. In theory, the robotic platform offers distinct advantages for technically demanding resections near the hepatic hilum and major vessels, as well as for vascular or biliary reconstructions. Its use has already been reported in complex scenarios such as surgery for hilar cholangiocarcinoma, living donor hepatectomy, and living donor liver transplantation [1,30]. Hu et al. suggested that minimally invasive surgery for hilar cholangiocarcinoma remains limited to carefully selected patients and is technically feasible only in highly experienced centers. Nonetheless, further refinements in surgical techniques and instrumentation are required to reduce morbidity and facilitate wider adoption of minimally invasive approaches for its treatment [31].

Based on our review, the age of patients varies greatly, reaching 88 years, demonstrating a relatively wide application of robotic surgery. The site most commonly associated with metastatic issues is the rectum, and the robot performs particularly well in minor hepatic resections. However, there is limited long-term data, although the encouraging results motivate us to increasingly use this surgical approach in the future. Many of the studies reviewed evaluated the feasibility of robotic-assisted surgery for the treatment of colorectal liver metastases (CRLMs), highlighting its beneficial role even in patients with multiple metastases or those who had undergone previous or simultaneous surgeries. Emerging imaging technologies [32] such as near-infrared (NIR) fluorescence with indocyanine green (ICG) may help offset the absence of tactile sensation in minimally invasive surgery and act as a valuable complement to intraoperative ultrasound (IOUS). Many laparoscopic and robotic platforms currently in clinical use are either compatible with or already integrated with NIR fluorescence imaging capabilities. The use of ICG fluorescence can offer surgeons real-time visualization of tumor boundaries during minimally invasive procedures for colorectal liver metastases, potentially enhancing the rate of complete oncologic resections. Achterberg et al. showed that ICG-guided imaging was linked to a higher frequency of margin-negative resections and led to intraoperative strategy modifications in over 25% of cases. Notably, the lack of ICG signal in the liver during parenchymal dissection was found to predict an R0 resection with 92% precision [10]. These findings indicate that ICG fluorescence can support intraoperative decision-making and improve oncologic outcomes by providing accurate real-time delineation of tumor margins. A limitation of our review is certainly the number of reviewed studies; however, few of them focused on the strictly surgical and robotic aspects. A key limitation of the available evidence is that most included series represent single-center experiences with explicit or implicit selection of patients considered suitable for a minimally invasive/robotic approach. Other limitations are related to data on the exact timing of liver resection relative to the colorectal procedure, perioperative systemic therapy (neoadjuvant or adjuvant chemotherapy), and the segmental location of liver metastases were inconsistently reported across the included studies. Similarly, reporting of postoperative complications varied; although pooled Clavien–Dindo grades are presented, many studies lacked detailed breakdowns of specific complications (e.g., biliary leak, POLF, reoperation). This considerable heterogeneity and incomplete reporting constitute an important limitation and affect the strength of the conclusions. Future prospects are definitely the continued advancements that are anticipated in both resection techniques and diagnostic tools. Technologies such as artificial intelligence—which is already being implemented in laparoscopy—augmented reality, intraoperative navigation, and hybrid operating rooms are expected to play an increasingly important role. These innovations support surgeons in managing complex anatomies that are not visible on the surface and often exhibit significant individual variation.

## 7. Conclusions

As research progresses and new tools become available for the treatment of CRLM, surgery will retain a predominant role. Robotic liver surgery is now a consolidated reality. Its application in the treatment of liver metastases from colorectal cancer is essential for the future. This minimally invasive approach involves treatment of the pathology either concurrently with or subsequent to the primary surgery, with encouraging outcomes. Robotic combined resection for colorectal cancer with liver metastases is technically achievable and appears to offer comparable oncologic outcomes to those of open or laparoscopic procedures. Further research is necessary to reinforce our conclusions, considering the relatively small number of studies included here. While robotic liver surgery is gaining wider adoption for selected indications, further studies are needed to assess its long-term surgical and oncologic outcomes, as well as its cost-effectiveness, in comparison with conventional laparoscopic and open techniques.

## Figures and Tables

**Table 1:** Characteristics of patients enrolled.

Patients	N	Age (Years)	BMI (kg/m^2^)	Primary Tumor Location Right ⟶ Left ⟶ Rectum	Liver Disease
**Navarro et al., 2019** **[9]**	**1** **2**	**59 (37–77)**	**24.9 ± 2.4**	**2 ⟶ 1 ⟶ 10**	**n/a**
Achterberg et al., 2024 [10]	201	65 (57–72)	n/a	40 ⟶ 65 ⟶ 96	n/a
McGuirk et al., 2021 [11]	2 8	62.5	22.4	7 ⟶ 7 ⟶ 14	n/a
Guadagni et al., 2020 [12]	20	66.1 ± 11.8	24.4 ± 2.7	n/a	n/a
Rocca et al., 2024 [13]	4 5	66.53 ± 12.67	25.95 ± 3.83	11 ⟶ 15 ⟶ 19	n/a
Machado et al., 2019 [14]	1	64	n/a	1	n/a
Kenary et al., 2025 [15]	1	78	n/a	1	n/a
Krishnamurthy et al., 2018 [16]	1	57	n/a	1	n/a
Wang et al., 2023 [17]	1	52	n/a	1	n/a
Marino et al., 2020 [18]	40	69.4 (38–79)	25.2 (18.5–28.8)	n/a	Hepatitis B 5 (12.5%), Hepatitis C 4 (10%), Cirrhosis 6 (15%)
Pesi et al., 2019 [19]	5 1	63 (31–81)	n/a	n/a	Cirrhosis 13 (25.5%)
Mehdorn et al., 2021 [20]	50	64.0 ± 12.3 (40–82)	27.4 ± 6.7 (19.5–40.4)	n/a	n/a
Winckelmans et al., 2023 [21]	2 10	age 5 years 92 20.4%, age < 75 years 34 19.2%	n/a	n/a	n/a
Rahimly et al., 2022 [22]	50	66.9 (10.5)	27.9 (5.0)	n/a	Cirrhosis Child A 10 (25.0) Child B 2 (5.0) Hepatic steatosis 14 (35.0) Hepatitis B 2 (5.0) Hepatitis C 1 (2.5)

## Data Availability

The datasets used and/or analyzed during the current study are available from the corresponding author on reasonable request.
